# Objective–Subjective Socioeconomic Status Configurations, Self-Acceptance, and Adolescent Prosocial Moral Reasoning: A Response Surface Analysis

**DOI:** 10.3390/bs16081367

**Published:** 2026-08-10

**Authors:** Xiaoming Li, Juan Zhang, Xiaoyun Zhao, Cheng Guo

**Affiliations:** 1 Faculty of Psychology, Southwest University, Chongqing 400715, China; 2 School of Foreign Languages, Guizhou University of Engineering Science, Bijie 551700, China; 3 School of Education Science, Huaiyin Normal University, Huai’an 223300, China; 4 School of Education, Huaibei Normal University, Huaibei 235000, China; zhaoxiaoyun1980@163.com

**Keywords:** adolescent moral reasoning, subjective socioeconomic status, objective socioeconomic status, response surface analysis, self-acceptance

## Abstract

**Background:** Objective and subjective socioeconomic status capture distinct aspects of adolescents’ socioeconomic experience, but their joint associations with prosocial moral reasoning and self-acceptance remain unclear. This study examined objective–subjective socioeconomic status configurations in relation to prosocial moral reasoning and self-acceptance, and assessed statistical indirect associations through self-acceptance. **Methods:** A cross-sectional survey was conducted with 1888 Chinese adolescents aged 12 to 18 years. Participants completed measures of objective socioeconomic status, subjective socioeconomic status, self-acceptance, and prosocial moral reasoning. Polynomial regression and response surface analysis were used, with indirect associations tested using 5000 bootstrap resamples. **Results:** The full polynomial regression model predicting prosocial moral reasoning was significant, but explained variance was small, and response surface parameters did not indicate significant direct congruence or incongruence effects. Socioeconomic status configurations were more clearly associated with self-acceptance. Self-acceptance was higher when objective and subjective socioeconomic status were both relatively high and when subjective socioeconomic status exceeded objective socioeconomic status. Self-acceptance was positively associated with prosocial moral reasoning, and bootstrap analyses showed significant but very small indirect associations through self-acceptance. **Conclusions:** Objective–subjective socioeconomic status configurations showed limited direct associations with overall prosocial moral reasoning but clearer associations with self-acceptance. Given the cross-sectional design and small effect sizes, the findings should be interpreted as modest statistical associations rather than evidence of causal mediation.

## 1. Introduction

Socioeconomic status (SES) is an important developmental context that shapes adolescents’ access to family resources, educational opportunities, social networks, and exposure to stressors (Bradley & Corwyn, 2002; Conger & Donnellan, 2007; McLoyd, 1998). In adolescent development, SES should not be understood only as an objective family condition. It is also a social position that young people perceive, interpret, and compare with others. Objective SES is usually measured by indicators such as parental education, occupation, and family economic conditions, whereas subjective SES refers to adolescents’ perceived family standing within a social hierarchy (Adler et al., 2000; Goodman et al., 2001; Kraus et al., 2012).

The distinction between objective and subjective SES is especially meaningful during adolescence, a period marked by heightened peer comparison, social evaluation, and identity formation (Harter, 2012; Goodman et al., 2007). Recent post-COVID and contemporary adolescent research further indicates that socioeconomic conditions continue to shape adolescents’ psychological adjustment, future orientation, and well-being (Fakkel et al., 2023; Rakesh et al., 2025). At the same time, subjective SES is not fully reducible to objective family socioeconomic indicators. Recent evidence shows that adolescents’ subjective SES is moderately associated with parent-reported and administrative SES indicators and can predict multiple well-being outcomes (Davisson et al., 2025). These findings suggest that objective SES and subjective SES capture related but distinct aspects of adolescents’ socioeconomic experience. Therefore, they should be examined jointly rather than treated as interchangeable indicators of the same background condition.

### 1.1. Objective and Subjective SES and Prosocial Moral Reasoning

Moral reasoning refers to how individuals make judgments about welfare, fairness, responsibility, rights, and social obligation (Turiel, 1983; Smetana, 2006; Killen & Smetana, 2015). Prosocial moral reasoning focuses more specifically on how individuals reason about helping, sharing, and responding to others’ needs (Carlo et al., 1992; Eisenberg et al., 2006). From a social reasoning developmental perspective, adolescents’ moral judgments are not formed in isolation from their social experiences. Instead, they are shaped by how young people understand group relations, social status, inequality, and responsibility (Rutland & Killen, 2015; Killen & Dahl, 2021).

Recent studies provide further support for connecting socioeconomic experience with adolescents’ moral cognition. For example, adolescents have been found to evaluate social class-based exclusion as morally wrong and to show increasing awareness of discrimination and unfair treatment with age (Gönül et al., 2023). Other recent work has shown that intergroup contact with peers from lower socioeconomic backgrounds can reduce exclusion based on social class (Gönül et al., 2024). Research on distributive fairness and educational inequality also suggests that children and adolescents consider structural barriers, nondiscrimination, and social equality when reasoning about resource allocation (Grütter et al., 2024). Recent Chinese evidence also indicates that prosocial moral reasoning develops within broader school and developmental contexts rather than as an isolated individual trait (Zhao et al., 2025). Together, these findings suggest that SES is not only a background demographic variable but also a meaningful social–developmental context for adolescents’ prosocial moral reasoning.

However, most previous studies have examined objective SES and subjective SES as separate predictors. This approach may overlook how structural socioeconomic resources and adolescents’ perceived social position combine within development. A configuration perspective may help address this limitation. Objective–subjective SES congruence refers to the extent to which adolescents’ perceived socioeconomic standing matches their family’s objective socioeconomic position. When both objective and subjective SES are high or low, adolescents may have a relatively consistent understanding of their social location. When the two are incongruent, the psychological meaning may differ by direction. Adolescents with high objective SES but low subjective SES may experience relative deprivation, upward comparison, or status dissatisfaction despite having more family resources. In contrast, adolescents with low objective SES but high subjective SES may hold a more positive interpretation of their social position, which could buffer some negative effects of objective disadvantage (Goodman et al., 2007; Quon & McGrath, 2014; Sweeting & Hunt, 2014; Bøe et al., 2019; Davisson et al., 2025). These different SES configurations may be relevant to prosocial moral reasoning because adolescents’ judgments about helping, fairness, and responsibility may depend not only on their actual resources but also on how they perceive their own position in relation to others.

### 1.2. The Role of Self-Acceptance

Self-acceptance is a core dimension of psychological well-being and refers to a positive and realistic attitude toward the self, including acceptance of one’s strengths, limitations, past experiences, and current circumstances (Ryff, 1989, 2013; MacInnes, 2006). Compared with global self-esteem, which emphasizes evaluation of self-worth, self-acceptance reflects a more integrated and less externally contingent form of self-regard (MacInnes, 2006; Ryff, 2013).

The relationship between SES and self-acceptance can be understood from the perspective of social comparison and self-development. During adolescence, young people increasingly evaluate themselves in relation to peers and social groups (Harter, 2012). If adolescents perceive their socioeconomic position as unfavorable, they may be more likely to experience dissatisfaction, status anxiety, or difficulty accepting their current circumstances. This may be especially evident when subjective SES is lower than objective SES, because adolescents may compare themselves with more advantaged peers and interpret their own position negatively. By contrast, when subjective SES is higher than objective SES, adolescents may maintain a more positive understanding of their social position, which may support a stronger sense of self-acceptance even under objective socioeconomic constraints (Goodman et al., 2007; Quon & McGrath, 2014; Bøe et al., 2019; Davisson et al., 2025). Thus, SES configurations may be associated with self-acceptance by reflecting the consistency between adolescents’ structural conditions and their subjective self-understanding.

Self-acceptance may also be associated with prosocial moral reasoning. Adolescents with higher self-acceptance may be less dominated by defensive self-protection, external approval, or status insecurity. This may allow them to pay more attention to others’ needs and to reason from the perspective of empathy, responsibility, and concern for others’ welfare (Eisenberg et al., 1991, 2006; van der Graaff et al., 2018; Hollarek & Lee, 2022). In contrast, adolescents with lower self-acceptance may be more sensitive to social evaluation and personal insecurity, which may make their moral reasoning more self-protective or approval-oriented. Therefore, self-acceptance provides a theoretically meaningful link between adolescents’ socioeconomic experiences and their prosocial moral reasoning.

### 1.3. The Present Study

The conceptual ordering examined in the present study was guided by developmental-context and family-process perspectives. Objective SES, reflected in parental education, occupation, and family economic conditions, represents a relatively distal structural context that is largely external to adolescents’ current self-related functioning and moral reasoning. These socioeconomic conditions may shape adolescents’ access to material and educational resources, exposure to stress, social networks, social comparison contexts, and opportunities to encounter and interpret issues involving need, inequality, and social responsibility (Bradley & Corwyn, 2002; Conger & Donnellan, 2007; Conger et al., 2010).

Subjective SES was conceptualized not as another purely distal condition but as adolescents’ appraisal of their socioeconomic position within family, school, and peer contexts. Although this appraisal may itself be influenced by adolescents’ personal characteristics, social awareness, and moral interpretations, it remains partly anchored in family socioeconomic conditions and salient comparison contexts (Goodman et al., 2007; Quon & McGrath, 2014; Bøe et al., 2019; Davisson et al., 2025). Empirical research therefore supports the component associations underlying the proposed model, although direct longitudinal evidence for the complete temporal ordering remains limited.

Within this framework, self-acceptance was positioned as a more proximal self-system correlate, and prosocial moral reasoning was treated as the focal social-cognitive outcome. This conceptual ordering was specified a priori as one theoretically plausible model rather than as an empirically established or uniquely correct temporal sequence. In particular, adolescents’ moral awareness and interpretations of inequality may also influence their subjective social status, and reverse and reciprocal relationships remain plausible.

Guided by this framework, the present study treated objective SES as a relatively distal structural context, subjective SES as adolescents’ appraisal of their socioeconomic position, self-acceptance as a more proximal self-system correlate, and prosocial moral reasoning as the focal social-cognitive outcome. Accordingly, the present study addressed the following research questions: Q1. Are objective SES, subjective SES, and their configurations associated with adolescents’ prosocial moral reasoning? Q2. Are objective SES, subjective SES, and their configurations associated with adolescents’ self-acceptance? Q3. Are objective–subjective SES configurations statistically associated with prosocial moral reasoning through their shared associations with self-acceptance? We expected that adolescents with higher matched levels of objective and subjective SES would show higher self-acceptance and prosocial moral reasoning. We also expected that the direction of objective–subjective SES incongruence would be meaningful. Specifically, subjective SES lower than objective SES may be associated with lower self-acceptance, whereas subjective SES higher than objective SES may be associated with relatively higher self-acceptance. Finally, we expected self-acceptance to be positively associated with prosocial moral reasoning and to be involved in statistical indirect associations between SES configurations and prosocial moral reasoning. Because the study used a cross-sectional design, the indirect associations were interpreted as statistical associations rather than causal mediation.

## 2. Methods

### 2.1. Participants and Procedure

This study used a cross-sectional survey design. A total of 2177 adolescents were recruited from junior and senior secondary schools in China. Before analysis, the dataset was screened for out-of-range values, invalid codes, and implausible responses. Cases with invalid or implausible responses on variables used in the main analyses were excluded. The final analytic sample consisted of 1888 adolescents with complete valid data on all study variables, including 992 males and 896 females. Participants ranged in age from 12 to 18 years (M = 14.58, SD = 2.06).

Prior to data collection, participants and their legal guardians were informed of the study’s purpose, voluntary participation, and confidentiality of responses. Informed consent was obtained before participation. Participants completed a standardized questionnaire battery assessing socioeconomic status, prosocial moral reasoning, self-acceptance, and related psychosocial variables in school settings under standardized instructions. All responses were anonymized before analysis.

### 2.2. Measures

#### 2.2.1. Objective and Subjective Socioeconomic Status

Objective socioeconomic status was measured using a demographic information questionnaire. Adolescents reported information on parental education, parental occupation, and family economic condition. These indicators were combined to form an objective SES score, represented by the variable OSES in the dataset. In the analytic sample, OSES scores ranged from 4 to 22, with higher scores indicating higher objective socioeconomic status.

Subjective socioeconomic status was measured using the MacArthur Scale of Subjective Social Status, adapted to assess adolescents’ perceived family social standing. Participants were asked to place their family on a social ladder ranging from 0 to 10, where higher positions represented higher perceived socioeconomic status. In the dataset, subjective SES was represented by the variable SSES, with higher scores indicating higher subjective socioeconomic status.

#### 2.2.2. Moral Reasoning

Adolescents’ prosocial moral reasoning was assessed using the Chinese version of the Prosocial Moral Reasoning Objective Measure (PROM; Carlo et al., 1992). The PROM is a self-report measure designed to assess adolescents’ reasoning in prosocial moral dilemmas (Lai et al., 2012). It was developed on the basis of Eisenberg’s prosocial moral reasoning framework and presents participants with hypothetical situations in which the protagonist’s own needs or desires conflict with the needs of others. Participants are asked to evaluate the importance of different reasons for the protagonist’s behavioral choice.

In the present study, moral reasoning was indexed by the PROM maturity index; higher scores indicated more mature prosocial moral reasoning. The PROM maturity index was used as the sole outcome variable in the present study. In the analytic sample, PROM maturity scores ranged from 2.31 to 3.45.

Previous studies have supported the reliability and validity of the PROM and its Chinese version among adolescents. Because the available analytic dataset contained the composite maturity index rather than raw item-level responses, internal consistency or scoring reliability for the PROM could not be recalculated from the current analytic file. Therefore, reliability information was reported based on the original scale or prior validation studies.

#### 2.2.3. Self-Acceptance

Self-acceptance was assessed using the Self-Acceptance Questionnaire developed by Cong and Gao (1999). The original questionnaire contains 16 items and consists of two dimensions: self-acceptance and self-evaluation. In the present study, the self-acceptance dimension was used. This dimension includes eight items and assesses the extent to which adolescents acknowledge, understand, and accept themselves, including both positive and negative aspects of the self.

Participants responded to each item on a 4-point Likert scale, with higher scores indicating greater self-acceptance. Negatively worded items were reverse-scored where appropriate, and item scores were summed to create a total self-acceptance score. In the present dataset, scores ranged from 8 to 32 in the analytic sample. In the present study, Cronbach’s alpha for the self-acceptance subscale was 0.86.

### 2.3. Data Analysis

Data analyses were conducted in several steps. First, descriptive statistics and bivariate correlations were calculated for the main study variables. Gender and age were included as covariates in the subsequent regression models because previous developmental research suggests that adolescents’ self-related functioning and moral reasoning may vary by demographic characteristics.

Polynomial regression combined with response surface analysis was used to examine the joint associations of objective SES and subjective SES with adolescents’ prosocial moral reasoning and self-acceptance. This approach was selected because the present study focused on the configuration between objective and subjective SES rather than the isolated effect of either variable. Simple difference scores were not used because they collapse two conceptually distinct predictors into a single index and cannot distinguish high–high congruence from low–low congruence or one direction of incongruence from the opposite direction. Polynomial regression and response surface analysis allow the independent, interactive, and curvilinear associations of objective and subjective SES to be estimated simultaneously, and they provide interpretable parameters along the line of congruence and the line of incongruence.

Because objective SES and subjective SES were measured on different original scales, both variables were standardized before the squared and interaction terms were created. Therefore, congruence and incongruence were interpreted as coordinated or opposing deviations in standardized objective and subjective SES rather than exact equality on the raw measurement scales. The polynomial regression model was specified as follows:

*Moral reasoning* = *b*_0_ + *b*_1_*OSES_z* + *b*_2_*SSES_z* + *b*_3_*OSES_z*^2^ + *b*_4_*OSES_z* × *SSES_z* + *b*_5_*SSES_z*^2^ + *gender* + *age* + *e*.

The same polynomial terms were also used to predict self-acceptance. Response surface parameters were calculated from the estimated polynomial coefficients. The slope and curvature along the line of congruence were estimated as a_1_ = b_1_ + b_2_ and a_2_ = b_3_ + b_4_ + b_5_, respectively. The slope and curvature along the line of incongruence were estimated as a_3_ = b_1_ − b_2_ and a_4_ = b_3_ − b_4_ + b_5_, respectively. A significant a_1_ indicates that the outcome changes when objective SES and subjective SES increase or decrease together. A significant a_3_ indicates that the outcome differs depending on the direction of incongruence between objective and subjective SES.

Self-acceptance was further examined in terms of statistical indirect associations between SES configurations and prosocial moral reasoning. In this model, the SES polynomial terms were used to predict self-acceptance, and self-acceptance was used to predict prosocial moral reasoning while controlling for the SES polynomial terms, gender, and age. Indirect associations were tested using 5000 bootstrap resamples. Percentile-based 95% bootstrap confidence intervals that did not include zero were interpreted as evidence of significant indirect associations. Because the study used a cross-sectional design, these indirect associations were interpreted as statistical indirect effects rather than evidence of causal mediation.

Because the main variables were partly collected through self-report questionnaires, common method bias should be considered. The study used several procedural controls, including anonymous administration, confidentiality assurances, and standardized instructions. Based on the available composite variables, an auxiliary single-factor diagnostic showed that the first unrotated component explained 34.32% of the total variance, which was below the commonly used 40% or 50% criterion. This finding suggests that common method bias was unlikely to fully account for the observed associations. Nevertheless, because complete item-level data were not available in the analytic file, this diagnostic should be interpreted cautiously, and common method bias cannot be entirely excluded. All analyses were conducted in R version 4.5.1 using RStudio.

## 3. Results

### 3.1. Preliminary Analyses

After data screening and the exclusion of invalid or implausible responses, the final analytic sample consisted of 1888 adolescents with valid data on gender, age, objective SES, subjective SES, self-acceptance, and the PROM maturity index. Descriptive statistics and bivariate correlations among the study variables are presented in Table 1. Gender and age were included as covariates in the subsequent regression models.

As shown in Table 1, objective SES was positively correlated with subjective SES, *r* = 0.29, *p* < 0.001, indicating that adolescents from higher objective socioeconomic backgrounds tended to report higher perceived socioeconomic status. Age was negatively correlated with objective SES, *r* = −0.28, *p* < 0.001, subjective SES, *r* = −0.20, *p* < 0.001, self-acceptance, *r* = −0.07, *p* < 0.01, and the PROM maturity index, *r* = −0.21, *p* < 0.001. These age-related correlations were interpreted descriptively and were controlled for in the subsequent regression models.

Subjective SES was positively associated with self-acceptance, *r* = 0.11, *p* < 0.001. In contrast, objective SES was not significantly associated with self-acceptance, r = 0.02, *p* > 0.05. Both objective SES and subjective SES were positively correlated with the PROM maturity index. However, the magnitudes were small, *r* = 0.06, *p* < 0.01, and *r* = 0.09, *p* < 0.001, respectively. Self-acceptance was also positively associated with the PROM maturity index, *r* = 0.13, *p* < 0.001. Overall, these preliminary results suggested modest associations among subjective socioeconomic status, self-acceptance, and adolescents’ prosocial moral reasoning.

### 3.2. Joint Associations of Objective and Subjective SES with Overall Moral Reasoning

Polynomial regression and response surface analysis were conducted to examine whether objective SES and subjective SES jointly predicted adolescents’ prosocial moral reasoning. Objective SES and subjective SES were standardized before creating the squared and interaction terms, and gender and age were included as covariates.

The full polynomial regression model predicting the PROM maturity index was statistically significant, with *R*^2^ = 0.046, adjusted *R*^2^ = 0.043, *F*(7, 1880) = 13.094, and *p* < 0.001. However, the explained variance was small, indicating that the overall predictive power of the model was limited. Therefore, the statistical significance of the full model should be interpreted cautiously, especially given the large sample size (Table 2).

At the coefficient level, age was a significant negative predictor of the PROM maturity index, with *B* = −0.010, *SE* = 0.001, *t* = −8.142, and *p* < 0.001, and subjective SES was a significant positive predictor, with *B* = 0.006, *SE* = 0.003, *t* = 2.258, and *p* = 0.024. Objective SES, the squared terms, and the objective SES × subjective SES interaction term were not significant predictors. These results suggest that the significance of the full model was mainly attributable to the covariate effect of age and the small linear effect of subjective SES, rather than to a strong objective–subjective SES configuration effect (Table 3).

**Figure 1:**
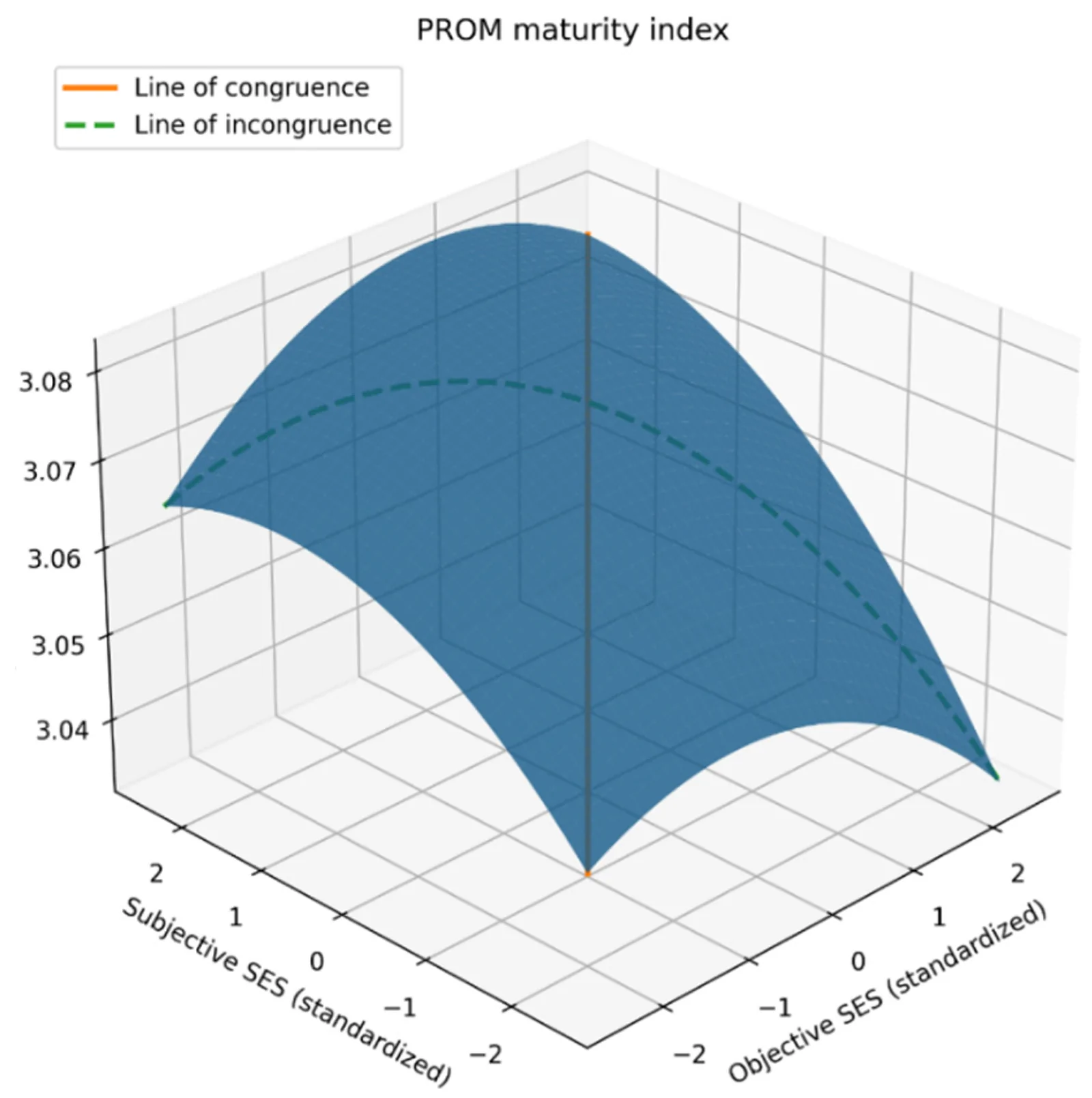
Response surface plot of objective SES and subjective SES predicting PROM maturity index.

The response surface parameters further supported this interpretation (see Figure 1). For the PROM maturity index, the slope along the line of congruence was not significant, with *a*_1_ = 0.006, *SE* = 0.003, *t* = 1.683, and *p* = 0.093, and the curvature along the line of congruence was also not significant, with *a*_2_ = −0.002, *SE* = 0.003, *t* = −0.718, and *p* = 0.473. These findings indicate that prosocial moral reasoning did not significantly increase or decrease as objective SES and subjective SES increased together.

Similarly, neither the slope along the line of incongruence, with *a*_3_ = −0.006, *SE* = 0.004, *t* = −1.498, and *p* = 0.134, nor the curvature along the line of incongruence, with *a*_4_ = −0.004, *SE* = 0.005, *t* = −0.908, and *p* = 0.364, reached statistical significance. Thus, there was no clear evidence that prosocial moral reasoning differed as a function of the direction or degree of discrepancy between objective and subjective SES. In other words, the direct configuration effect of objective and subjective SES on prosocial moral reasoning was not supported in the present data.

Taken together, although the full regression model was statistically significant, the response surface analysis did not provide strong evidence for direct congruence or incongruence effects of objective and subjective SES on prosocial moral reasoning. The predicted response surface was relatively flat, suggesting that SES configurations were not strongly associated with PROM maturity directly (Table 4).

### 3.3. Statistical Indirect Associations Through Self-Acceptance

**Figure 2:**
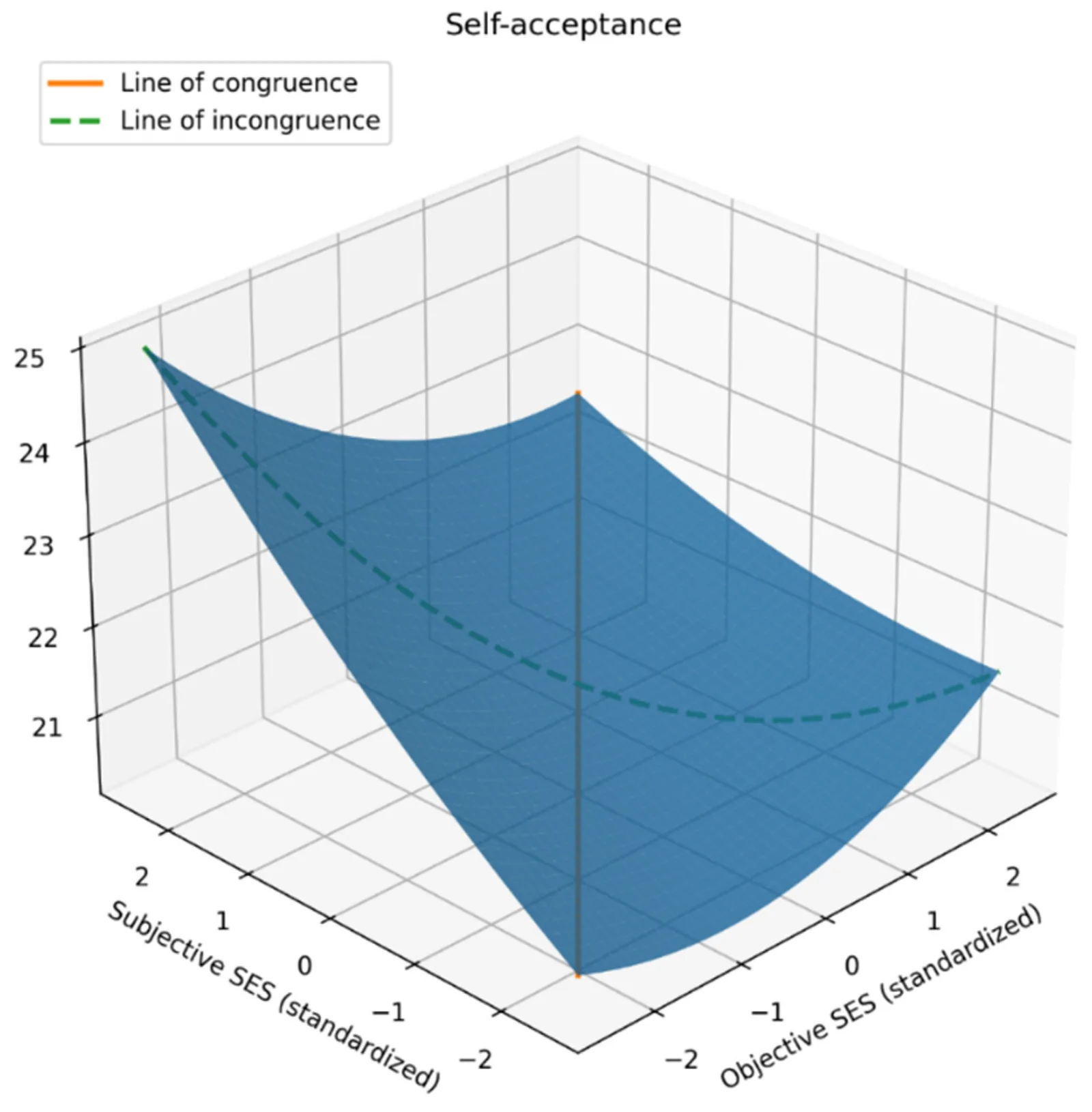
Response surface plot of objective SES and subjective SES predicting self-acceptance.

Self-acceptance was then examined in terms of statistical indirect associations between objective–subjective SES configurations and prosocial moral reasoning (see Figure 2). The polynomial regression model predicting self-acceptance was statistically significant, with *R*^2^ = 0.020, adjusted *R*^2^ = 0.016, *F*(7, 1880) = 5.449, and *p* < 0.001. Although the explained variance was small, the response surface parameters showed clearer patterns for self-acceptance than for prosocial moral reasoning.

At the coefficient level, subjective SES was a significant positive predictor of self-acceptance, with *B* = 0.542, *SE* = 0.117, *t* = 4.620, and *p* < 0.001. Gender and age were also significant predictors, with lower self-acceptance observed among female adolescents, with *B* = −0.479, *SE* = 0.221, *t* = −2.161, and *p* = 0.031, and older adolescents, with *B* = −0.134, *SE* = 0.056, *t* = −2.368, and *p* = 0.018. Objective SES, the squared terms, and the objective SES × subjective SES interaction term were not significant.

The response surface parameters indicated a significant positive slope along the line of congruence, with *a*_1_ = 0.383, *SE* = 0.146, *t* = 2.617, and *p* = 0.009. This suggests that self-acceptance tended to be higher when objective SES and subjective SES were both relatively high than when both were relatively low. The curvature along the line of congruence was not significant, with *a*_2_ = 0.029, *SE* = 0.149, *t* = 0.194, and *p* = 0.846, indicating that this association was primarily linear rather than curvilinear.

The slope along the line of incongruence was also significant, with *a*_3_ = −0.701, *SE* = 0.189, *t* = −3.703, and *p* < 0.001. Given the coding of the response surface parameters, this negative slope indicates that adolescents whose subjective SES was relatively higher than their objective SES reported higher self-acceptance than adolescents whose objective SES was relatively higher than their subjective SES. The curvature along the line of incongruence was not significant, with *a*_4_ = 0.303, *SE* = 0.216, *t* = 1.401, and *p* = 0.161, suggesting that the incongruence pattern was directional rather than curvilinear.

Finally, self-acceptance was positively associated with the PROM maturity index after controlling for gender, age, objective SES, subjective SES, and their polynomial terms, with *B* = 0.002, *SE* = 0.001, *t* = 4.684, and *p* < 0.001. Bootstrap analyses based on 5000 resamples indicated significant indirect associations through self-acceptance. The indirect association along the line of congruence was significant, with indirect *a*_1_ = 0.00094 and 95% CI [0.00018, 0.00198], and the indirect association along the line of incongruence was also significant, with indirect *a*_3_ = −0.00171 and 95% CI [−0.00312, −0.00060].

These indirect effects were statistically significant but very small in magnitude. Therefore, the findings should not be interpreted as evidence of a strong mediation effect. Rather, they suggest that objective–subjective SES configurations were more clearly associated with self-acceptance than with prosocial moral reasoning directly, and that self-acceptance was involved in modest statistical indirect associations between SES configurations and adolescents’ prosocial moral reasoning.

## 4. Discussion

The present study examined whether objective and subjective SES jointly related to adolescents’ prosocial moral reasoning and whether self-acceptance was involved in statistically indirect associations. The findings should be interpreted cautiously and in a differentiated way. The direct response surface for PROM maturity was relatively flat, whereas the response surface for self-acceptance showed clearer, although still small, effects. This pattern suggests that objective–subjective SES configurations may be more closely related to adolescents’ self-related adjustment than to broad prosocial moral reasoning directly. This interpretation is consistent with prior evidence that objective and subjective SES are related but nonredundant aspects of adolescents’ socioeconomic experience, and that subjective SES is often especially relevant to adolescents’ psychological adjustment and well-being (Goodman et al., 2007; Quon & McGrath, 2014; Sweeting & Hunt, 2014; Bøe et al., 2019; Davisson et al., 2025). However, given the small effect sizes and cross-sectional design, the findings should be understood as modest statistical associations rather than evidence of strong developmental mechanisms.

### 4.1. SES Configurations and Prosocial Moral Reasoning

Contrary to the expectation that objective–subjective SES congruence would be directly associated with prosocial moral reasoning, neither the congruence nor the incongruence parameters reached significance. This result indicates that objective–subjective SES configurations were not strong direct predictors of adolescents’ overall prosocial moral reasoning. It is important to distinguish the significance of the full polynomial regression model from the significance of theoretically meaningful response surface parameters. The full model tested whether the complete set of predictors, including covariates and polynomial terms, explained variance in PROM maturity. In contrast, the response surface parameters tested whether the estimated surface showed meaningful slopes or curvatures along the line of congruence or incongruence (Edwards & Parry, 1993; Shanock et al., 2010; Humberg et al., 2019; Nestler et al., 2019). In the present study, the latter evidence was weak.

This finding qualifies, rather than rejects, the relevance of SES for adolescent moral development. Socioeconomic conditions may shape adolescents’ developmental opportunities, social comparison contexts, and exposure to inequality. Nevertheless, prosocial moral reasoning is also influenced by multiple social-cognitive and motivational factors, such as empathy, perspective taking, moral identity, parenting, peer norms, and school climate (Eisenberg et al., 2006; Aquino & Reed, 2002; Hardy & Carlo, 2011; van der Graaff et al., 2018; Hollarek & Lee, 2022). Therefore, adolescents with higher or more favorable SES configurations should not be assumed to show more mature moral reasoning simply because of their socioeconomic position.

One possible explanation is that the PROM maturity index measures general prosocial reasoning rather than moral judgments specifically involving social class, inequality, exclusion, or distributive justice. Recent studies have shown that adolescents reason about social class-based exclusion, educational inequality, distributive fairness, and structural barriers in morally meaningful ways (Gönül et al., 2023, 2024; Grütter et al., 2024). Thus, SES configurations may be more strongly related to domain-specific moral judgments about inequality than to a broad index of prosocial moral reasoning. Future research should therefore use scenarios involving resource distribution, class-based exclusion, and structural disadvantage to examine whether socioeconomic experience becomes more salient when moral issues directly involve inequality.

### 4.2. SES Configurations and Self-Acceptance

Compared with the model predicting prosocial moral reasoning, the response surface for self-acceptance showed clearer patterns. Self-acceptance increased when objective SES and subjective SES increased together. This suggests that adolescents with both relatively favorable family resources and favorable perceived status may hold a more positive and integrated view of themselves. This finding is consistent with the conceptualization of self-acceptance as a core dimension of psychological well-being, involving acceptance of one’s strengths, limitations, experiences, and current circumstances (Ryff, 1989, 2013; MacInnes, 2006). It also aligns with research showing that subjective SES is associated with adolescent mental health, school functioning, and well-being beyond objective socioeconomic indicators (Goodman et al., 2007; Quon & McGrath, 2014; Bøe et al., 2019; Davisson et al., 2025).

The direction of incongruence also mattered for self-acceptance. Adolescents whose subjective SES exceeded their objective SES reported higher self-acceptance than those whose objective SES exceeded their subjective SES. This pattern can be understood through social comparison theory and relative deprivation perspectives. Subjective SES is shaped not only by objective family resources but also by adolescents’ comparisons with salient reference groups, such as classmates, peers, and school communities (Festinger, 1954; Goodman et al., 2001, 2007; Kraus et al., 2012). When adolescents perceive their status as lower than their objective resources might suggest, they may experience relative disadvantage, upward comparison, dissatisfaction, or status insecurity (Sweeting & Hunt, 2014; Quon & McGrath, 2014; Vidal & Wissow, 2023). These experiences may make it more difficult to form a stable and accepting view of the self.

By contrast, subjective SES exceeding objective SES may reflect a more favorable interpretation of one’s social position. This does not necessarily mean that adolescents misperceive their family background. Rather, it may indicate stronger belonging, recognition, adaptive coping, or positive identity construction within their immediate social environment. Developmental theories emphasize that adolescence is a period in which identity formation, peer comparison, and self-evaluation become especially salient (Erikson, 1968; Harter, 2012). Therefore, relatively high subjective SES may support self-acceptance by helping adolescents integrate their social circumstances into a more coherent and positive self-view.

However, this result should not be interpreted as evidence that subjective SES is always more important than objective SES, or that objective disadvantage is psychologically harmless. Objective SES remains important because it reflects material resources, educational opportunities, family constraints, and exposure to stressors (Bradley & Corwyn, 2002; Conger & Donnellan, 2007; McLoyd, 1998; Rakesh et al., 2025). The present finding suggests only that perceived status may be particularly relevant to self-related adjustment during adolescence.

### 4.3. Self-Acceptance as a Statistical Indirect Association

Self-acceptance was positively associated with PROM maturity after controlling for gender, age, objective SES, subjective SES, and their polynomial terms. Bootstrap analyses further indicated significant indirect associations through self-acceptance. Thus, although SES configurations were not directly associated with prosocial moral reasoning in a clear response surface pattern, they were statistically associated with PROM maturity through self-acceptance. This pattern helps clarify why self-acceptance was included in the model. Self-acceptance is not merely a general adjustment variable; it is a self-system construct that may connect adolescents’ interpreted status experiences with their capacity to consider others’ needs in prosocial dilemmas (Ryff, 1989, 2013; MacInnes, 2006).

This interpretation is consistent with theories linking the self-system to moral motivation and moral judgment. Moral identity theory suggests that moral concerns become more motivationally powerful when they are integrated into one’s self-understanding (Blasi, 1984; Aquino & Reed, 2002; Hardy & Carlo, 2011). Although self-acceptance is not equivalent to moral identity, adolescents who accept themselves and their circumstances may be less preoccupied with defensive self-evaluation, status insecurity, or external approval. This may allow more psychological resources for perspective taking, empathy, and concern for others’ welfare, which are closely related to prosocial moral reasoning and prosocial behavior (Eisenberg et al., 1991, 2006; van der Graaff et al., 2018; Hollarek & Lee, 2022).

Nevertheless, the indirect associations were very small. Therefore, the findings should not be interpreted as evidence of a strong mediation effect. A more appropriate interpretation is that SES configurations may show a modest self-related pathway to prosocial moral reasoning through self-acceptance. This cautious interpretation is necessary because the direct response surface for PROM maturity was not significant and the explained variance in the models was limited. Small effects are common when broad developmental outcomes are predicted by distal contextual variables, but they should be interpreted in proportion to their magnitude (Funder & Ozer, 2019).

The cross-sectional design further limits causal interpretation. Although the model was theoretically specified with SES configurations predicting self-acceptance and self-acceptance predicting prosocial moral reasoning, the temporal order among these variables cannot be established. Cross-sectional indirect effects cannot confirm mediation processes or rule out reverse and reciprocal associations (Maxwell & Cole, 2007; Maxwell et al., 2011; MacKinnon, 2008). Longitudinal studies are needed to test whether SES configurations predict later self-acceptance and whether self-acceptance subsequently predicts changes in moral reasoning.

### 4.4. Contributions and Practical Implications

This study makes three main contributions. First, it shows that objective–subjective SES configurations do not necessarily translate into strong direct differences in broad prosocial moral reasoning. This helps avoid reducing moral maturity to socioeconomic advantage. Social reasoning developmental theory emphasizes that moral judgments are shaped by the coordination of welfare, fairness, rights, group processes, and contextual knowledge rather than by a single background factor (Turiel, 1983; Smetana, 2006; Rutland & Killen, 2015; Killen & Dahl, 2021). The present findings are consistent with this view.

Second, the study demonstrates that the same SES configurations were more clearly associated with self-acceptance. This finding extends previous work on objective and subjective SES by showing that their configuration may matter for adolescents’ self-related adjustment. Prior studies have shown that subjective SES predicts adolescent health, psychological adjustment, and well-being beyond objective SES indicators (Goodman et al., 2007; Quon & McGrath, 2014; Sweeting & Hunt, 2014; Bøe et al., 2019; Davisson et al., 2025). The present study adds that the match or mismatch between structural resources and perceived status may be relevant to self-acceptance.

Third, the study illustrates the methodological value of polynomial regression and response surface analysis. A significant full regression model can coexist with nonsignificant theoretically meaningful surface parameters. Therefore, researchers should not equate the significance of the overall model with support for congruence or incongruence hypotheses (Edwards & Parry, 1993; Shanock et al., 2010; Humberg et al., 2019; Nestler et al., 2019).

Practically, the findings suggest that schools should pay attention to adolescents’ subjective status experiences and self-acceptance, but these implications should be stated cautiously. Visible status comparison, material-based peer ranking, and exclusionary peer climates may undermine students’ self-related adjustment, especially during adolescence when peer evaluation and social identity are salient (Harter, 2012; Goodman et al., 2007; Vidal & Wissow, 2023). School practices that strengthen belonging, reduce harmful comparison, and support self-acceptance may provide a more favorable psychological context for prosocial reflection (Ryan & Deci, 2000; Osterman, 2000; Ryff, 2013). However, such practices should not replace efforts to address objective socioeconomic disadvantage.

### 4.5. Limitations and Future Directions

Several limitations should be noted. First, the cross-sectional design prevents causal inference and does not establish temporal ordering. Reverse and reciprocal associations are particularly plausible for subjective SES. Adolescents with greater sensitivity to fairness, inequality, and social responsibility may interpret and report their socioeconomic position differently, and their moral reasoning may also be related to how they understand and accept themselves. Future longitudinal research, including cross-lagged panel designs, should compare the hypothesized conceptual ordering with reverse and reciprocal models.

Second, the main variables were based on self-report, which may introduce common method variance. The PROM also relies on hypothetical moral dilemmas and self-reported reasoning, and the study did not include a social desirability measure. Future research should include multiple data sources, such as behavioral tasks, teacher or parent reports, peer reports, interview-based moral reasoning assessments, and social desirability controls (Podsakoff et al., 2003, 2012).

Third, the explained variance was small, especially for PROM maturity. Future research should examine additional factors, such as moral identity, empathy, perspective taking, parenting style, peer acceptance, school belonging, perceived discrimination, classroom moral climate, and exposure to moral dialogue (Eisenberg et al., 2006; Hardy & Carlo, 2011; Rutland & Killen, 2015; Killen & Dahl, 2021; Hollarek & Lee, 2022).

Finally, because objective and subjective SES were measured on different scales and then standardized, the line of congruence should be interpreted as coordinated deviations from the sample mean rather than strict raw-score equality. Future studies could use more comparable SES indicators and examine whether SES configurations are more strongly related to moral reasoning in scenarios involving inequality, resource distribution, exclusion, or social class.

## 5. Conclusions

This study examined the joint associations of objective and subjective SES with adolescents’ prosocial moral reasoning and examined statistically indirect associations through self-acceptance. The direct response surface results did not show significant congruence or incongruence effects for PROM maturity, suggesting that SES configurations were not strong direct predictors of broad prosocial moral reasoning. However, SES configurations were more clearly associated with self-acceptance, and self-acceptance was positively associated with moral reasoning. These effects were small and non-causal, but they suggest that the psychological significance of SES configurations may lie less in their direct association with overall moral reasoning and more in their association with adolescents’ self-acceptance. The study therefore contributes to adolescent developmental research by showing how structural socioeconomic conditions and perceived status can be examined jointly without reducing moral development to socioeconomic background alone.

## Figures and Tables

**Table 1:** Descriptive statistics and correlations among study variables.

Variable	M	SD	1	2	3	4	5
1. Gender	1.47	0.50	—				
2. Age	14.58	2.06	0.05 *	—			
3. Objective SES	11.17	4.37	−0.07 **	−0.28 ***	—		
4. Subjective SES	5.49	1.73	−0.01	−0.20 ***	0.29 ***	—	
5. Self-acceptance	21.49	4.81	−0.05 *	−0.07 **	0.02	0.11 ***	—
6. PROM	3.07	0.11	−0.04	−0.21 ***	0.06 **	0.09 ***	0.13 ***

**Note.** N = 1888. Values are Pearson correlation coefficients. * *p* < 0.05, ** *p* < 0.01, *** *p* < 0.001.

**Table 2:** Model fit for polynomial regression models.

Outcome	*N*	*R* ^2^	Adjusted *R*^2^	*F*	df1	df2	*p*
PROM maturity index	1888	0.046	0.043	13.094	7	1880	<0.001
Self-acceptance	1888	0.02	0.016	5.449	7	1880	<0.001

**Table 3:** Polynomial regression coefficients predicting PROM maturity and self-acceptance.

Predictor	PROM Maturity				Self-Acceptance			
	*B*	SE	*t*	*p*	*B*	SE	*t*	*p*
Intercept	3.239	0.02	161.529	<0.001	24.018	0.881	27.26	<0.001
Gender	−0.007	0.005	−1.360	0.174	−0.479	0.221	−2.161	0.031
Age	−0.010	0.001	−8.142	<0.001	−0.134	0.056	−2.368	0.018
Objective SES_z	−0.000	0.003	−0.153	0.879	−0.159	0.122	−1.303	0.193
Subjective SES_z	0.006	0.003	2.258	0.024	0.542	0.117	4.62	<0.001
Objective SES_z^2^	−0.002	0.003	−0.662	0.508	0.12	0.121	0.99	0.322
Objective SES_z × Subjective SES_z	0.001	0.003	0.368	0.713	−0.137	0.121	−1.129	0.259
Subjective SES_z^2^	−0.002	0.001	−1.095	0.274	0.046	0.065	0.705	0.481

**Note.** Objective SES_z and Subjective SES_z indicate standardized objective and subjective socioeconomic status.

**Table 4:** Response surface parameters and indirect effects through self-acceptance.

Outcome/Effect	Estimate	SE	*t*	*p*	95% CI
**PROM maturity index**					
*a*_1_: Congruence slope	0.006	0.003	1.683	0.093	—
*a*_2_: Congruence curvature	−0.002	0.003	−0.718	0.473	—
*a*_3_: Incongruence slope	−0.006	0.004	−1.498	0.134	—
*a*_4_: Incongruence curvature	−0.004	0.005	−0.908	0.364	—
**Self-acceptance**					
*a*_1_: Congruence slope	0.383	0.146	2.617	0.009	—
*a*_2_: Congruence curvature	0.029	0.149	0.194	0.846	—
*a*_3_: Incongruence slope	−0.701	0.189	−3.703	<0.001	—
*a*_4_: Incongruence curvature	0.303	0.216	1.401	0.161	—
Self-acceptance → PROM maturity index	0.002	0.001	4.684	<0.001	—
Indirect *a*_1_ via self-acceptance	0.00094	—	—	—	[0.00018, 0.00198]
Indirect *a*_3_ via self-acceptance	−0.00171	—	—	—	[−0.00312, −0.00060]

**Note.** Indirect effects were estimated using 5000 bootstrap resamples. Confidence intervals are percentile-based 95% bootstrap confidence intervals. *a*_1_ and *a*_2_ represent the slope and curvature along the line of congruence, respectively. *a*_3_ and *a*_4_ represent the slope and curvature along the line of incongruence, respectively.

## Data Availability

All data and materials are available. Please contact the author to request.
